# Molecular Interactions of Fluoroquinolone Antibiotics with Lipid Membranes

**DOI:** 10.1021/acs.langmuir.5c04836

**Published:** 2025-12-02

**Authors:** D. Ralph, A. Goode, V. Yeh, J. M. A. Blair, P. Williams, B. B. Bonev

**Affiliations:** † School of Life Sciences, 6123 University of Nottingham, QMC, Nottingham NG7 2UH, U.K.; ‡ Department of Microbes, Infection and Microbiomes, Institute of Microbiology and Infection,University of Birmingham, Birmingham B15 2TT, U.K.

## Abstract

Levofloxacin is a broad-spectrum fluoroquinolone antibiotic in clinical use that targets DNA gyrase in the cytosol. It is used in systemic applications via oral or intravenous route, and its pharmacokinetics and access to its molecular targets are strongly influenced by interactions with cellular membranes. We used NMR and MD simulations to investigate the physical state of levofloxacin in solution and its interactions with lipid membranes, assessing the role of membrane charge and antibiotic concentration. Using zwitterionic DOPC and negatively charged DOPC/DOPG lipid membranes, we observe concentration-dependent self-association of levofloxacin in solution below its solubility limits and association with lipid membranes with a preference for negatively charged bilayers. Below the solubility limit, levofloxacin solutions that appear clear contain self-associated molecular assemblies in fast exchange equilibrium with a monomeric soluble population. In the presence of lipid membranes, this equilibrium is shifted in favor of a membrane-associated population with preference for negative lipids. MD simulations show levofloxacin condensation in solution and fractional membrane insertion, which suggest the presence of a molecular population embedded in the lipid bilayer, coexisting with self-associated levofloxacin molecular “droplets” in exchange with a solvated population.

## Graphical abstract

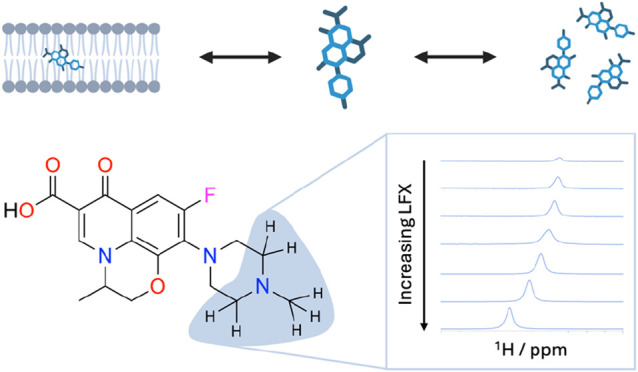

## Introduction

Since their introduction, antibiotics have revolutionized modern medicine, dramatically reducing morbidity and mortality in human and veterinary healthcare worldwide.^1^ Fluoroquinolones are a well-established class of broad-spectrum agents that exert antibacterial activity by inhibiting DNA gyrase and topoisomerase IV, enzymes critical for DNA replication and repair.^2^ While the pharmacodynamics of fluoroquinolones is well characterized, their interactions with lipid membranes, which influence cellular uptake, distribution, and efficacy, remain less well understood at the molecular level. Elucidating the molecular details of fluoroquinolone-membrane interactions is critical for understanding drug partitioning at biological interfaces and guiding the development of antibiotics with enhanced membrane permeability and efficacy.

Levofloxacin, a third-generation fluoroquinolone, is widely prescribed due to its broad antimicrobial spectrum across Gram-positive and Gram-negative bacterial species, high oral bioavailability, and favorable pharmacokinetics.^3^ Despite being functionally active within the bacterial cytoplasm, levofloxacin is only moderately lipophilic with reported logP values of −0.4,^4^ 0.6,^5^ and 1.27,^6^ suggesting limited membrane binding with possible slight aqueous phase preference.^7^ This raises fundamental questions about its molecular organization in solution, partitioning into biological membranes, and ability to traverse cell membranes and enter cells via passive diffusion. The levofloxacin molecule is zwitterionic at physiological pH, which suggests different possibilities for its interactions with neutral and negative membranes. In addition, the presence of fluorine and an aromatic ring offer a good reporter system for NMR characterization. As a representative fluoroquinolone, levofloxacin serves as a tractable and clinically relevant model for establishing baseline interactions that help us understand the molecular interactions of other fluoroquinolone antibiotic compounds.^8^

Biological membranes serve as both barriers and regulatory interfaces for antibiotic passage. In Gram-negative bacteria, the outer and inner membranes control the influx of small molecules through a combination of structural lipids and embedded proteins.^9^ For levofloxacin to reach its intracellular target enzymes, it needs to cross both membranes for therapeutic efficacy, with factors that affect influx including hydrophobicity, molecular size, and charge.^10^ Fluoroquinolones cross the outer bacterial membranes by passive diffusion via porins, primarily OmpC and OmpF.^11^ The inner membrane is largely composed of phospholipids, such as phosphatidylethanolamine (PE), phosphatidylglycerol (PG), and cardiolipin, and has an overall negative charge.^12,13^ In this study, we use DOPC and DOPG as model lipids to mimic zwitterionic and anionic bilayer environments, respectively, to investigate how membrane surface charge modulates the localization, binding orientation, and dynamic behavior of levofloxacin in the presence of lipid bilayers. We use zwitterionic phosphatidylcholine lipids instead of phosphatidylethanolamine, the predominant lipid species in bacterial membranes, because the methylated phosphatidylcholine, like phosphatidylglycerol, forms structurally stable lipid bilayers in the absence of membrane proteins and a cytoskeletal support. Maintaining bilayer integrity is important, as drug-lipid interactions can influence membrane structure and stability, fluidity, and can impact drug bioavailability.^14^ We hypothesize here that levofloxacin can exist in multiple physicochemical states, as monomers in solution, self-associated aggregates, or membrane-bound species, depending on concentration and bilayer composition. Understanding such a distribution that governs membrane-permeation mechanisms is important for understanding and predicting drug efficacy and pharmacokinetics.

Here, we study the state of levofloxacin in solution and its interactions with model lipid membranes to elucidate how bilayer composition influences fluoroquinolone behavior. Proton and fluorine solution NMR spectroscopy, and phosphorus and carbon solid-state NMR, were combined with molecular dynamics (MD) simulations to examine the behavior of levofloxacin in aqueous and lipid environments. NMR provides atomic-level insights into drug dynamics and self- and membrane association, while MD simulations offer a detailed, time-resolved view of drug-drug and drug-lipid interactions under different physicochemical conditions and membrane charge. Molecular details from the ternary levofloxacin/lipid/water system offer a better understanding of antibiotic distribution, bioavailability, and molecular mechanism, as well as of the potential adaptive changes in the bacterial envelope that may lead to increased tolerance to fluoroquinolones. These characteristics are crucial in guiding the molecular design of existing and next-generation antibiotics with optimized intracellular targeting and resistance-evasion capabilities.

## Materials and Methods

### Reagents

Levofloxacin hemihydrate, deuterium oxide (D_2_O, 99.9% D), and methanol (99.9% pure) were purchased from Sigma-Aldrich (St. Louis, MO, USA). Lipids, DOPC (1,2-dioleoyl-*sn*-glycero-3-phosphatidyl choline), and DOPG (1,2-dioleoyl-*sn*-glycero-3-phosphatidyl glycerol) were purchased from Avanti Polar Lipids (Alabaster, AL, USA) at >99% purity and used without further purification. DOPC was used due to its tendency to form stable bilayers and is an inert molecule with a neutral, zwitterionic headgroup. Main transition temperatures of DOPC and DOPG are −16.5 and −18 °C, which ensures membrane fluidity at the temperature range of this study.^15–17^ DOPG was used to introduce negative charges in membranes at a 1:3 molar ratio to DOPC, the latter ensuring membrane phase stability.

### Liposome Preparation

Multilamellar vesicles (MLVs) and large unilamellar vesicles (LUVs) were prepared as described previously.^18,19^ DOPC powder (20 μmol) or DOPC/DOPG mixtures were dissolved in 3:1 methanol/chloroform, and the solvent was removed by rotary evaporation. All compound solutions were adjusted to pH 7.5 with either HCl or NaOH and added to the lipid film. Multilamellar vesicles (MLVs) were prepared by hydrating the lipid films followed by five cycles of freeze–thawing between liquid nitrogen and a 40 °C water bath.^20^ Large unilamellar vesicles (LUVs) were prepared by 11-passage extrusion of the hydrated lipid suspensions through 400 nm polycarbonate filter membranes^21^ using an Avanti extruder (Avanti Polar Lipids). Levofloxacin stock solutions and LUVs were stored at 4 °C until use. The chemical structures of levofloxacin and lipids used in this study are detailed in Figure 1.

**Figure 1:**
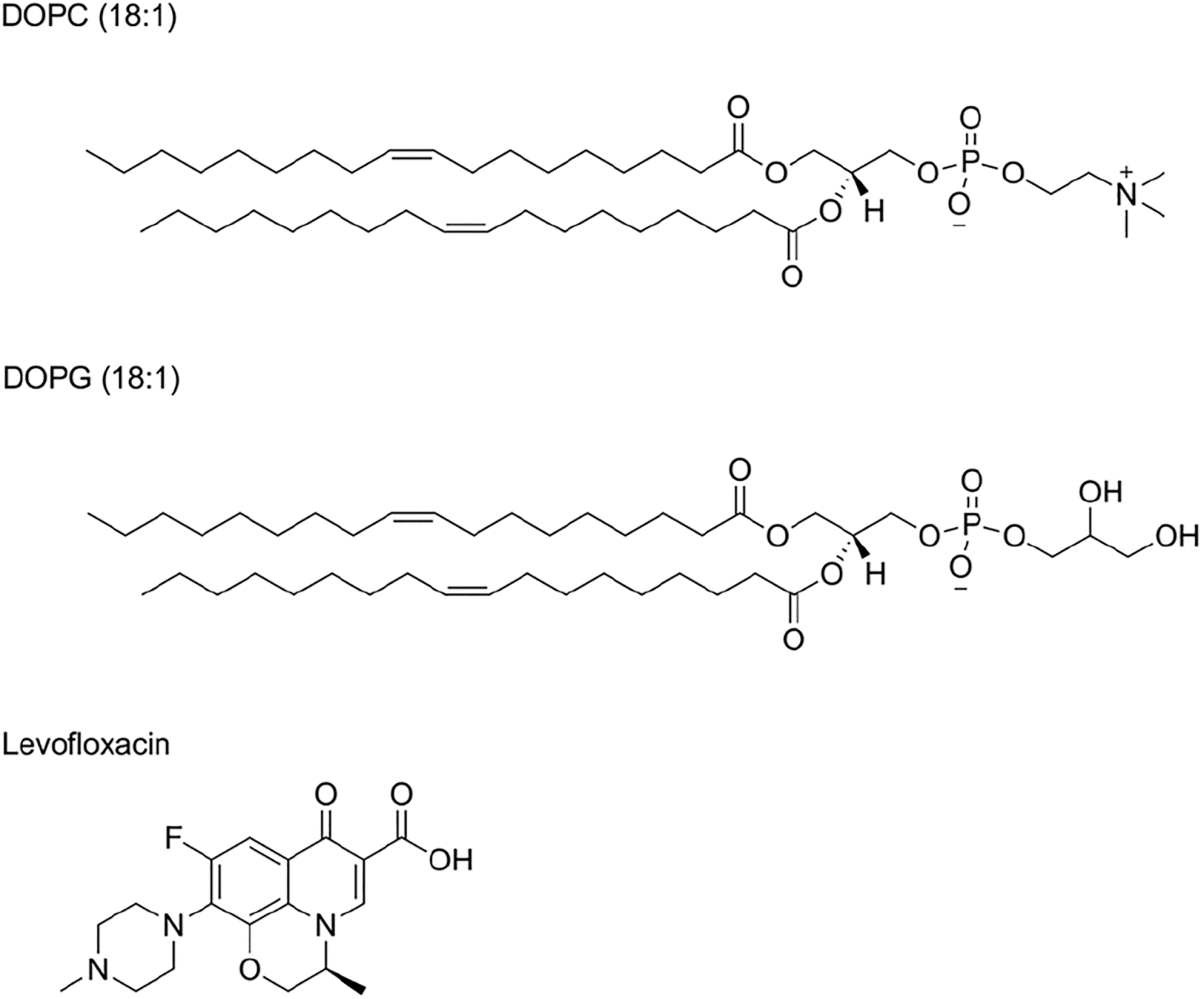
Chemical structure of levofloxacin, and lipids used in vitro and in silico: DOPC (18:1) and DOPG (18:1).

### Solution NMR

Samples for ^1^H and ^19^F NMR spectroscopy were prepared by dissolving levofloxacin hemihydrate in D_2_O at concentrations of 0.5, 1, 2, 5, 10, 20, and 40 mM. All ^1^H spectra were referenced to internal standard tetramethylsilane (TMS) at 0 ppm, and the NMR measurements were carried out on a Bruker AVANCE III spectrometer operating at 400 MHz proton frequency. Proton spectra were acquired following direct excitation with a 90° pulse width of 8 μs, acquisition time of 2.5 s without decoupling, relaxation delay of 1.0 s, and 64 scans were averaged for each FID. The ^19^F NMR spectra were acquired at 376 MHz following direct excitation with a 90° pulse of 10 μs, acquisition time of 2.0 s, relaxation delay of 1.5 s, and 64 scans were averaged for each FID. Spectra were processed with 0.1 Hz Lorentzian broadening.

### Solid-State NMR

All solid-state NMR experiments were performed on a Varian 400 MHz VNMRS widebore spectrometer equipped with a 4 mm T4 MAS NMR probe. Temperature was regulated using balanced heating/vortex tube cooled gas flow, and the measured values were corrected for known heating due to MAS and RF.^22,23^ All ^31^P spectra were referenced externally to 10% H_3_PO_4_ at 0 ppm, and ^13^C spectra were referenced externally to adamantane CH_2_ at 37.54 ppm. Phosphorus-31 wideline NMR was carried out at a frequency of 161.82 MHz and at a temperature of 20 °C using the Hahn echo sequence with 100 kHz π/2 and π-pulses separated by 12 μs interpulse and preacquisition delays. Spectra were acquired with a 25 ms acquisition time with a recycle delay of 5 s and with 1024 transients averaged to obtain each NMR free induction decay (FID) and processed with 80 Hz Lorentzian line broadening.

Inversion recovery was used under 5 kHz MAS to investigate ^31^P longitudinal relaxation with delay times varying between 10 ms and 1.5 s between inversion π-pulse and the observation π/2 pulse. Spectra were recorded with 50 ms acquisition time under a 60 kHz SPINAL-64 ^24^ decoupling scheme with a recycle delay of 9 s to exceed 5-fold ^31^P T_1_ values in membranes.^18^ Relaxation times T_1_ were calculated by assuming a single exponential relaxation mechanism.

High-resolution ^13^C CP MAS NMR was done at a MAS frequency of 5 kHz and at a temperature of 5 °C. A 120 kHz ^1^H excitation pulse was followed by 3.5 ms of 45 kHz Harmann-Hahn contact for magnetization transfer to ^13^C. Spectra were acquired under a 60 kHz SPINAL-64 ^24^ decoupling over 125 ms acquisition time by averaging 8192 transients per FID. The recycle delay was set to 3.5 s to exceed five times the proton T_1_ (∼0.5 s), and all spectra were processed with 15 Hz Lorentzian line broadening in ACDLabs 2024.

### Dynamic Light Scattering

Dynamic Light Scattering (DLS) was used to monitor the hydrodynamic diameter and polydispersity index (PDI) of the prepared liposome samples. Measurements were performed using a Malvern Zetasizer Nano ZS equipped with a 4 mW He–Ne laser operating at a wavelength of 633 nm. The scattering angle was fixed at 173° (backscatter). Samples were diluted 1000-fold in particle-free deionized water to avoid multiple scattering effects. For particle size and polydispersity, each measurement was performed at 25 °C and repeated in triplicate.

### Molecular Dynamics Simulations

MD simulations were carried out, as described previously.^26^ The levofloxacin structure was prepared from SMILES and parametrized using Ligand Modeler module of CHARMM-GUI and system assembly was done in the Multicomponent Assembler module.^27–29^ Atomistic MD simulations were carried out using NAMD^26,30^ on a Supermicro server equipped with multi-GPGPU NVIDIA K80 vector processors and on the HPC Midlands+ Tier 2 cluster.

Lipid patches of 50 × 50Å size were constructed from DOPC and DOPC/DOPG 3:1 and hydrated in 150 mM KCl buffer at pH 7.5 containing 100 antibiotic molecules positioned initially in the aqueous phase with ionic balance adjusted to achieve zero net charge of the system. All systems were equilibrated under fixed volume NVT ensemble conditions and at 310.15 K, and production runs were carried out isobarically as NPT ensembles using a Langevin piston to a total of 500 ns trajectory duration. Molecular visualization and trajectory analysis were done using UCSF Chimera.^31^

## Results and Discussion

We investigate levofloxacin self-association and its interactions with membranes using solution and solid state NMR spectroscopy, and atomistic MD simulations. High-resolution NMR provides experimental details from the molecular interactions and their impact on molecular conformation in the antibiotic molecules, observed during antibiotic self-assembly and membrane interactions, while MD simulations inform on conformational dynamics of the antibiotic-membrane system and offer a molecular view of the interactions.

### Levofloxacin Self-Association in Solution

We hypothesize that in the presence of hydrated membranes, levofloxacin attains an equilibrium of coexisting multimers, monomers in the aqueous phase, and a membrane-embedded population. To investigate the effect of concentration on the self-association of levofloxacin, we used solution NMR to follow changes in chemical shift and resonance broadening over a range of levofloxacin concentrations between 0.5 and 40 mM. Toward the lower end of the range, the concentration of levofloxacin notably impacts the signal/noise ratio, which remains sufficient for the purpose of this analysis and did not require longer acquisition. A solution study of fluoroquinolones including ciprofloxacin, norfloxacin, and enoxacin has shown that chemical shifts can also change significantly with varying concentrations.^32^ Levofloxacin solubility in water has been reported from 17 mg/mL (47 mM) to 73–108 mg/mL (202–299 mM), or at high pH, up to 272 mg/mL (753 mM), and for the racemate, ofloxacin, above 54.2 mg/mL (150 mM).^30,33^ The concentration range investigated here is below the reported solubility limits for levofloxacin, which suggests that any concentration-dependent chemical shift changes may reflect self-association to small oligomeric clusters.

To investigate self-association of levofloxacin with increasing concentration, we acquired ^1^H and ^19^F NMR from levofloxacin in D_2_O at a pD of 7.4 over a concentration range between 0.5 mM and 40 mM, and followed changes in isotropic chemical shifts, CS_iso_, from individual proton environments and fluorine (Figure 2). The greatest shift change was observed for H2, which moved significantly upfield with an increasing concentration of levofloxacin. Most protons showed upfield shifts, except protons H5–11, which shifted downfield along with the fluorine atom. No crossover between resonances was observed from 0.5 to 40 mM levofloxacin, suggesting consistent self-association behavior without exchange between distinct conformers on the NMR time-scale.

**Figure 2:**
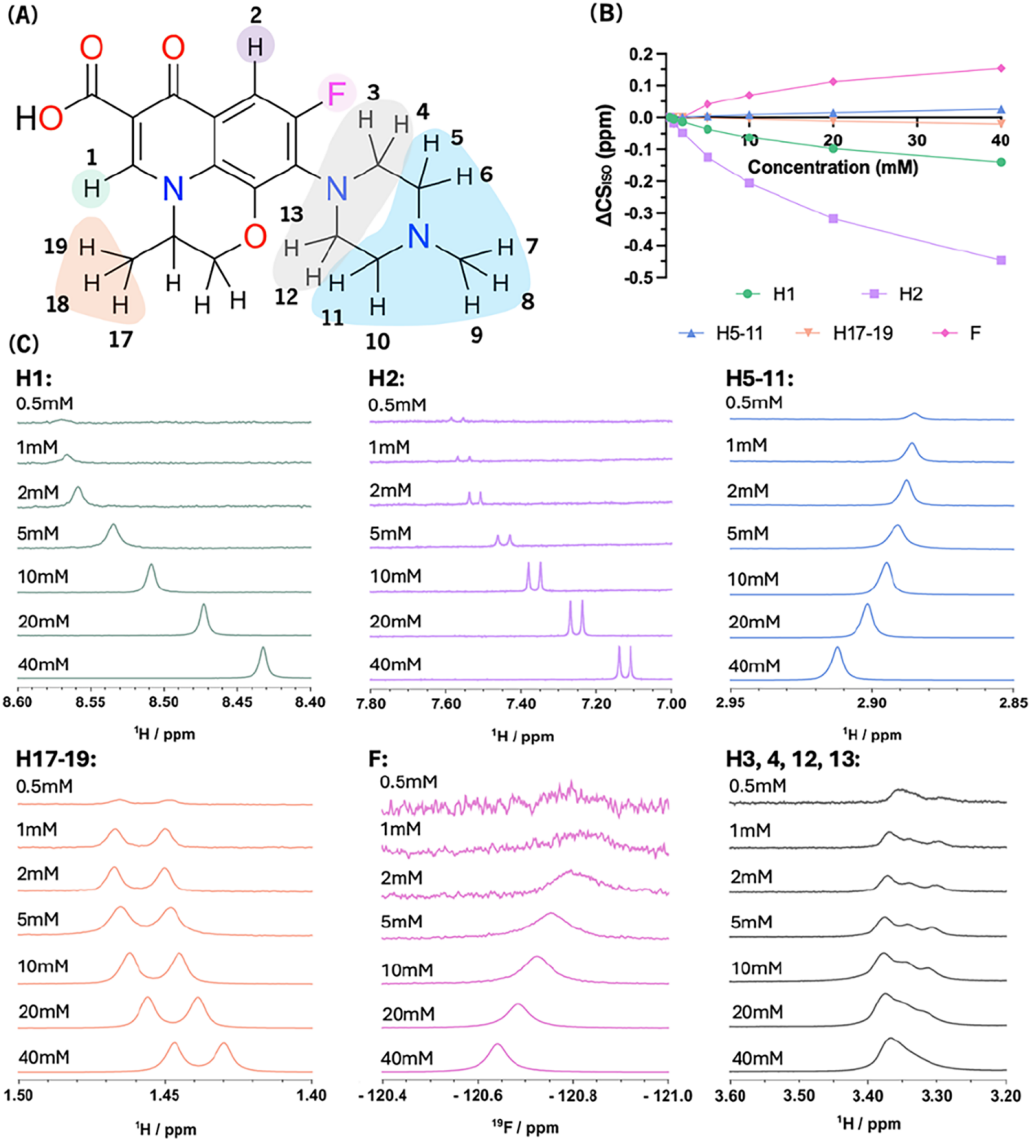
Concentration-dependent chemical shift changes of ^1^H and ^19^F resonances in the NMR spectra of levofloxacin. (A) Chemical structure of levofloxacin with protons highlighted in colors corresponding to the NMR spectra in section (C). (B) Levofloxacin concentration dependence of the deviation in CS_iso_ from its value at 0.5 mM for selected protons. (C) Changes in ^1^H NMR resonance shifts from individual protons of levofloxacin (panel (A): H1, H2, H5–11, H17–19, F, and H3, 4, 12, 13) over a concentration range from 0.5 to 40 mM. The spectra are color-coded to the corresponding protons highlighted in section (A).

Both ^1^H and ^19^F NMR spectra of levofloxacin in D_2_O reveal concentration-dependent chemical shift changes for several resonances. The direction and magnitude of these shifts vary between nuclei, suggesting that specific regions of the molecule are differentially affected by changes in the molecular environment on increasing levofloxacin concentration. These observations are consistent with an equilibrium between two states in fast exchange on the NMR time-scale. On the one hand, we have monomeric levofloxacin, characterized by an asymptotic approach of CS_iso_ toward the 0.5 mM values, and on the other, multimolecular clusters, in which levofloxacin molecules are likely to form ring-stacks that lead to more pronounced changes in CS_iso_ of ring protons and fluorine (Figure 2). Notably, protons such as H2, which exhibit the largest upfield shift, reveal regions that become increasingly buried or shielded during self-association, potentially due to π–π stacking or exclusion from the solvent. In contrast, protons that shift downfield or remain unchanged are likely solvent-exposed or less involved in intermolecular contacts. These trends allow us to infer which parts of the molecule participate directly in self-association interfaces. In this context, protons on the aromatic ring and near CC­(π) bonds shift upfield.^34,35^ Methyl piperazine protons 5–11 are primarily deshielded due to solvent effects, while H3, 4, 12, and 13 piperazine protons are less accessible by solvent molecules and outside the equatorial ring currents from the aromatic ring, showing an attenuated response to levofloxacin concentration. The fluorine CS_iso_ shows concentration-dependent deshielding, similar to the solvated piperazine protons 5–11. In contrast to piperazine equatorial proton 1, which is approximately 1.03 Å away from its carbon, the longer C–F bond in fluorobenzene is approximately 1.35 Å, and the equatorial ring current effects yield dominant solvent effects.

Previous studies have reported concentration-dependent chemical shift changes in small molecules such as benzene derivatives, initially attributed to ring current effects from aromatic systems.^36^ However, similar effects have been observed in nonaromatic systems, including in solvents like DMSO,^36^ suggesting that these chemical shift variations are not solely due to π–π interactions or aromatic ring currents but reflect a solvent contribution, as well.

To gain further insights into the molecular details of the levofloxacin state in the presence of membranes, we carried out atomistic MD simulations from 100 × 100 Å membrane patches in the presence of 100 solvated levofloxacin molecules (Figure 6). The initially solvated and dispersed levofloxacin molecular system coalesced into clusters and subsequently into a large droplet in aqueous solution, which exchanged rapidly with a small monomeric, solvated population. Within these clusters, individual levofloxacin molecules adopt a range of orientations and degrees of solvent exposure. Contacts observed in the MD trajectories include stacking interactions between the fluoroquinolone cores and close packing of hydrophobic regions such as the methyl piperazine groups. These interactions are consistent with NMR observations that less accessible protons, such as H2, experience increased shielding and undergo upfield shifts, while more solvent-accessible protons (e.g., H5–11) are deshielded or chemical shifts remain largely unchanged in the piperazine protons 3, 4, 12, and 13. Together, the NMR and MD data support a model, in which levofloxacin self-associates into loosely packed, nonuniform assemblies that exchange rapidly with solution. The exchange is fast (ns) on the NMR time-scale, and the observed CS_iso_ values reflect the weighted average of the two populations. Asymptotic values for each environment are obtained toward 0.5 and 40 mM solutions for monomeric and multimeric states, respectively.

A primary contributor to downfield shifts observed in NMR spectroscopy is the reduction in solvent shielding effects. Hydrogen bonding or dipole–dipole interactions with solvent molecules can increase the electron density around specific nuclei in polar environments, resulting in shielding from the external magnetic field causing upfield resonance shifts. At higher levofloxacin concentrations, self-association into molecular complexes reduces solvent exposure and leads to decreased shielding and downfield shifts. This is observed in functional groups including −OH or −NH, which undergo deshielding when transitioning from nonpolar or self-associated environments due to the reduction in hydrogen bonding and reduced solvent interactions.^37–39^ Similarly, self-association of levofloxacin in water appears to restrict hydrogen bonding with the solvent, highlighting the methylpiperazine group as a key hydrated moiety contributing to solubility alongside the oxopiperidine carboxylate moiety. By contrast, the aromatic core appears to be less accessible to solvent apart from the fluorine.

In polar solvents, dielectric stabilization of the electron cloud increases shielding by reducing the local magnetic field experienced by the nuclei, while apolar environments or self-association lead to downfield shifts in proton resonances.^38,39^ We use this effect to highlight molecular facets in levofloxacin that are solvated differentially on self-association or membrane insertion.

### Levofloxacin Interactions with Membranes

In all of its antimicrobial applications, levofloxacin encounters lipid membranes, which it has to cross before reaching its molecular target in the cytosol. To understand in molecular detail how levofloxacin molecules interact with lipid bilayers and potentially traverse the membrane, we use ^1^H and ^19^F NMR to follow concentration-dependent changes in levofloxacin NMR resonances in the presence of zwitterionic DOPC and acidic DOPC/DOPG mixed lipid membranes. Phosphatidylcholines are common in eukaryotic cells, while phosphatidylglycerol is a major membrane component in bacteria. PC is added to PG in our model to increase membrane stability and modulate headgroup electrostatic repulsion. A mixed environment of inert and negative target membranes exists in the sites of bacterial infections.

We acquired ^1^H and ^19^F NMR spectra from 0.5 to 40 mM levofloxacin in D_2_O (pD = 7.4) following hydration of preformed lipid films of DOPC or DOPC/DOPG (3:1). Both ^1^H and ^19^F spectra revealed lipid-specific concentration-dependent chemical shift changes in the presence of membranes (Figure 3). We use three groups of reporters on the levofloxacin molecular environment: aromatic side H2 and F; dihydropyridinone H1 and methyl protons H17–19 of dihydro oxazine; and methyl piperazine protons H5–11.

**Figure 3:**
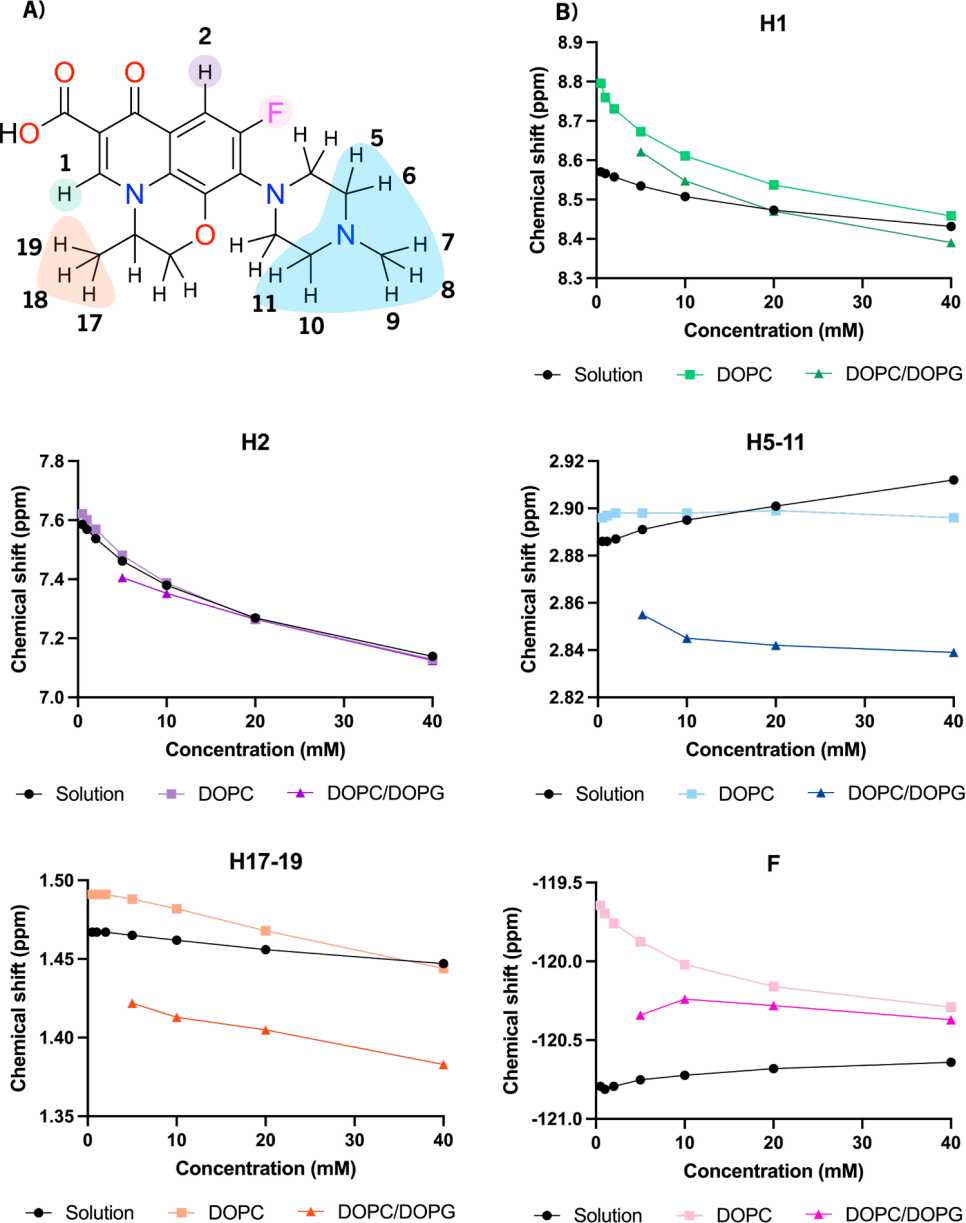
^1^H and ^19^F NMR chemical shifts of levofloxacin in solution acquired over a concentration range from 0.5 to 40 mM in the presence of zwitterionic and negative lipid membranes. (A) Molecular structure of levofloxacin highlighting molecular segments with concentration-dependent NMR chemical shifts. (B) Dependence of NMR chemical shifts in levofloxacin on concentration (0.5 to 40 mM). Concentration-dependent NMR shifts are shown from levofloxacin in solution and in the presence of either DOPC or DOPC/DOPG liposomes.

In the presence of either lipid system, quinolone aromatic proton H2 exhibited a monotonic concentration-dependent chemical shift change (Figure 3), similar to the observation in solution (Figure 3). With DOPC membranes, CS_iso_ followed closely the trend observed in solution, which indicates that the H2 solvation remains dominated by self-association at higher concentrations of levofloxacin. In the presence of negatively charged DOPG in membranes, the H2 proton appeared more shielded, which suggests a shift toward a more protected location of the aromatic proton H2. At a higher concentration, multimeric state in solution plays a determinant role and chemical shifts converge to the same value in solution and in the presence of membranes. Observation of a single resonance points to the coexistence of three environments in fast exchange-monomer, multimer, and a membrane-associated state.

The quinolone fluorine was deshielded on the addition of either type of lipid membrane. At the lowest concentration of 0.5 mM levofloxacin, the effect was more pronounced in neutral DOPC than in negative DOPC/DOPG membranes, with the resonances converging to the same chemical shift at about 20 mM levofloxacin and remaining deshielded compared to solution (Figure 3). In DOPC, the fluorine signal shifted downfield from −120.8 ppm in solution to −119.6 ppm with DOPC liposomes, which recovered upfield toward asymptotic −120.3 ppm at higher levofloxacin concentrations, while in the presence of negative DOPC/DOPG liposomes, the fluorine resonance shifted to −120.4 ppm and remained relatively unchanged at higher concentrations of levofloxacin. This indicates a stronger preference of levofloxacin for negatively charged membranes compared to zwitterionic membranes, the latter showing a shift to a membrane-associated state only at higher levofloxacin concentrations. The presence of either membrane type leads to preferential membrane association of levofloxacin and a shift away from the multimeric solution state to a common type of membrane-embedded environment.

At 0.5 mM levofloxacin, methyl piperazine protons H5–11 (Figure 3A) showed minor deshielding in DOPC membranes compared to solution, while in the presence of negative DOPC/DOPG membranes, the resonances underwent a slightly stronger upfield shift, revealing a preference for a more shielded environment (Figure 3). In DOPC, the resonance shifts remained near 2.9 ppm across all levofloxacin concentrations, with very little concentration-dependent variation. In DOPC/DOPG, H5–11 resonances shifted slightly upfield compared to solution, from 2.88 to 2.86 ppm, moving further to 2.84 ppm at 20 and 40 mM levofloxacin. The observed small upfield shift reflects a shielding effect due to deeper insertion or an altered electronic environment near the bilayer interface. In negatively charged membranes, proton H5–11 chemical shifts attain an asymptotic value above 10 mM, which indicates a stable equilibrium with preference for the membrane environment. By contrast, levofloxacin in solution shows further change in CS_iso_ toward 40 mM solution values. We conclude that levofloxacin is in equilibrium that favors a membrane-associated state with a different orientation in negative and zwitterionic membranes, shifted away from the molecular complex in solution.

At the lowest concentration of 0.5 mM levofloxacin, H1 protons were deshielded compared to solution in both lipids but more prominently in DOPC. At higher concentrations, the multimeric chemical shifts dominate and converge to the same value in solution and in the presence of membranes. Observation of a single resonance points to the coexistence of three environments in fast exchange-monomer, multimer, and a membrane-associated state. Dihydrooxazine methyl protons H17–19 are also slightly deshielded in zwitterionic DOPC membranes and at higher levofloxacin concentration showed CS_iso_ coalescing with solution values, which indicates weak association with membranes and shift toward multimeric state in solution at 40 mM levofloxacin. In the presence of negative DOPC/DOPG membranes, protons H17–19 were in a more shielded environment, with shielding increasing at higher levofloxacin levels. This indicated a decided preference of dihydrooxazine methyl protons H17–19 for the negative membrane environment and a shift in equilibrium in this direction. The concentration dependence trend in CS_iso_ parallels levofloxacin with DOPC membranes, but the equilibrium in that case is closer to the solution multimeric state. Protons H17–19 and H1 are away from the quinolone aromatic ring and are less affected by π-π stacking. Chemical shift changes in their resonances reflect environmental changes, such as solvation and membrane incorporation. Vinyl proton H1 is close to the carboxyl group, which remains solvated and draws electron density and dominates its chemical shift. Methyl protons H17–19 offer better insights into changes in solvation with monotonic concentration-dependent drift of CS_iso_ revealing a change in the levofloxacin position and orientation in membranes as membrane population increases.

### Membrane Incorporation of Levofloxacin

To understand better the impact of levofloxacin incorporation in membranes, we followed changes in membrane lipid organization using wideline ^31^P and ^13^C MAS ssNMR. Wideline ^31^P NMR informs on phase stability and slow lateral excursions of lipid molecules in the presence of amphipathic molecules;^26,40,41^ spectra from hydrated DOPC and DOPC/DOPG 3:1 liposomes without and with levofloxacin are shown in supplement Figure S1. The DOPC spectra without and with levofloxacin follow a classical powder distribution^43^ dominated by effective chemical shift anisotropy of 46 ppm, dominated by fast (∼GHz) axial lipid rotation. The subtle deviation from proper spherical distribution in the DOPC spectrum is reduced in the presence of levofloxacin, and we see a subtle increase in intensity near the isotropic center, consistent with a closer to spherical distribution and reduced lateral freedom of lipids due to a subtle increase in membrane rigidity. The mixed lipid system broadly shows a powder distribution with a significantly increased lateral mobility. This is seen as smearing of the 90° and 0° edges without affecting the overall effective chemical shift anisotropy (Figure S1). The presence of levofloxacin further increases the magnitude of lateral molecular excursions, as the antibiotic insertion into the membrane affects unequally lipid packing within the bilayer. A minor, broad distribution near lipid CS_iso_ indicates the onset of a small population with faster mobility, likely due to local effects on membrane curvature, associated with levofloxacin insertion.

To understand the impact of levofloxacin on fast lipid dynamics (∼GHz), we studied ^31^P longitudinal relaxation using inversion recovery under MAS. The observed T_1_ in DOPC was 780 ms, and it remained unaffected by levofloxacin. As DOPC T_1_ at 20 °C is near minimum,^18^ subtle changes in lipid dynamics may not impact T_1_ to a significant measure. Longitudinal relaxation times for DOPC and DOPG were shorter in the mixed lipid system, at 460 and 470 ms, respectively. This is reflective of reduced axial rotation rates associated with increased lateral excursions (librations). Addition of levofloxacin leads to an increase in T_1_ for DOPC to 570 ms and more notably in DOPG to 630 ms (Supporting Table ST1), indicative of preferential association of levofloxacin with DOPG.

We used high-resolution ^13^C MAS NMR under cross-polarization, CP, to distinguish membrane-incorporated levofloxacin from levofloxacin remaining in solution.^26,41^ Natural abundance ^13^C CP MAS NMR, acquired from DOPC or DOPC/DOPG 3:1 without and with levofloxacin are shown in Figure 4. CP NMR requires magnetization transfer from ^1^H to ^13^C and within the condensed ring structure of levofloxacin, many carbons lacking directly attached protons are not visible (Figure 4) in contrast to direct ^13^C excitation (Figure S2). However, observed levofloxacin resonances in the ^13^C CP MAS spectra reflect a uniquely membrane-incorporated antibiotic as CP is ineffective in solution. Spectral assignment is included in Supporting Table ST2. In the spectra from both DOPC membranes and DOPC/DOPG 3:1 mixed lipid membranes, we observe levofloxacin resonances, which confirms the existence of a membrane-incorporated levofloxacin population. Using the levofloxacin resonance intensities normalized to the lipid C9=C10 intensity (shared by DOPC and DOPG), we estimate the molar levofloxacin/lipid ratio in DOPC to be 1/16, while in the lipid mixture, it is 1/3. While these ratios are subject to the assumption that CP efficiency is similar for lipid and levofloxacin, the relative CP efficiency for levofloxacin is comparable in both bilayers, and we conclude that we have roughly five times more levofloxacin in the mixed lipid system than in DOPC alone. Considering each sample comprises 20 μmol lipid and 40 μmol levofloxacin, we estimate 3% levofloxacin incorporation into DOPC and 17% in the mixed lipid system with the rest remaining in solution.

**Figure 4:**
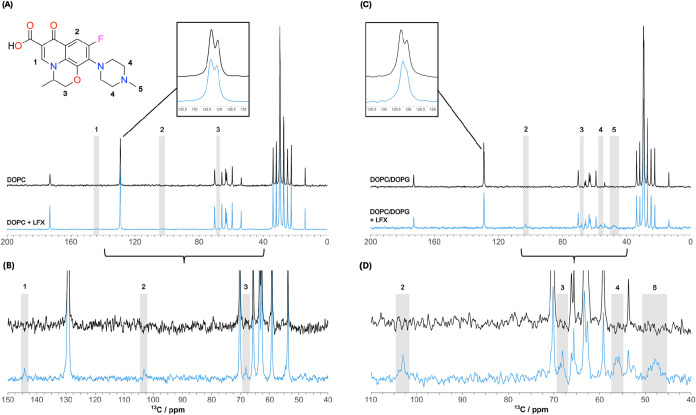
Carbon-13 cross-polarization MAS NMR spectra from lipid membranes without and with levofloxacin. Spectra from (A, B) hydrated DOPC, and (C, D) DOPC/DOPG (3:1) membranes without (black) or with 40 mM levofloxacin (blue) at 5 kHz MAS at 20 °C. Full spectra are shown in (A, C) with regions containing levofloxacin resonances zoomed in (B, D). Insets show carbon numbering for levofloxacin resonance assignment also shown in the spectra and expanded lipid C9=C10 resonances near 129 ppm.

We explore the location of levofloxacin within membranes by following changes in the lipid CS_iso_ on the addition of antibiotic. On visual inspection, Figure 4 reveals consistency in spectral resolution from C9=C10 DOPC chain carbons without or with levofloxacin (Figure 4, inset), while the addition of levofloxacin to mixed DOPC/DOPG bilayers selectively affects resolution in these resonances (Figure 4, inset). This offers direct evidence of the proximity of levofloxacin location to the lipid double bond in the mixed lipid membrane.

As we have shown previously, proximity to ring structures affects ^13^C shifts in lipid membranes.^45^ The ^13^C CP MAS NMR CS_iso_ values from DOPC and DOPC/DOPG mixed lipid membranes without and with levofloxacin are summarized in Table ST2 and assigned as previously reported.^23^ The differences between the CS_iso_ values before and after levofloxacin addition are shown for each resolved carbon moiety in Figure 5. In DOPC membranes, the most significant changes are observed for carbonyl, backbone, and terminal chain resonances, which indicates levofloxacin location within this region. Significant shift changes at the acyl chain terminal groups are consistent with proposed excursions of the acyl chain toward the backbone and headgroup region in lipid bilayers^46^ but can also indicate different subpopulations of membrane-embedded levofloxacin. In mixed lipid DOPC/DOPG membranes, the effects of levofloxacin are most pronounced in the headgroup resonances, as well as at the chain double bond, indicating headgroup localization of levofloxacin in the presence of negatively charged lipid, as well as the possibility of changes in headgroup packing resulting from deeper association with the membrane interior.

**Figure 5:**
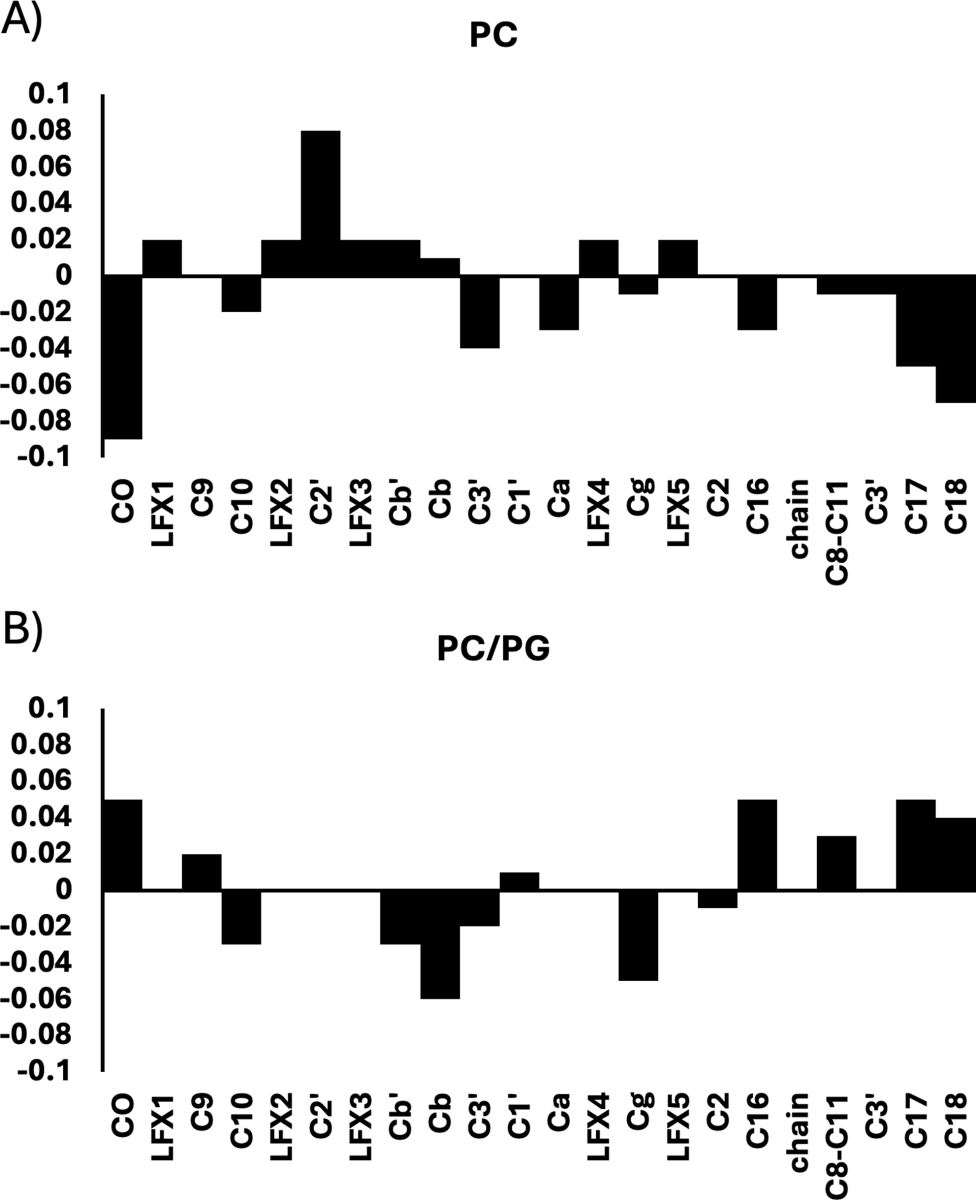
Levofloxacin-induced changes in ^13^C isotropic chemical shifts, CS_iso_, of membrane lipids from the CP MAS NMR spectra of Figure 4. (A) DOPC and (B) DOPC/DOPG showing more pronounced changes in CO and backbone region, as well as in lipid terminal groups; and in the headgroup region, respectively.

### MD Simulations of Levofloxacin in the Presence of Lipid Membranes

To understand how levofloxacin interacts with lipid membranes at the molecular level, we used atomistic MD simulations from 100 × 100 Å neutral and negatively charged lipid membranes, hydrated in the presence of 100 solvated levofloxacin molecules. Models of zwitterionic DOPC form stable bilayer structures in aqueous systems, as do DOPC/DOPG mixtures at a 1:3 molar ratio, which also present negatively charged membrane surfaces. Infection sites present both PC-rich host membranes and PG-rich bacterial surfaces, and selective insertion into PG-rich membranes is desirable for preferential delivery to the bacterial topoisomerase cytosolic targets.

Fluoroquinolones including levofloxacin and ciprofloxacin have been suggested to exhibit slight lipophilic properties.^47^ Using atomistic MD simulations, we follow the evolution of 100 levofloxacin molecules positioned in the aqueous phase near a simulated membrane patch. By the end of the 500 ns trajectory for the DOPC membrane, four antibiotic molecules integrated into the DOPC bilayer, while the rest rapidly coalesced (<100 ns) into a droplet within the aqueous phase and the system reached equilibrium (Figure 6A). Broadly, the 4% levofloxacin partitioning into membranes agrees with the NMR estimate of 3% molecular partitioning into DOPC membranes. The levofloxacin molecules that partition into the membrane did not exchange with the aqueous phase, while we did observe some droplet fragmentation and some monomer release from the droplet. Such self-association likely arises from intermolecular hydrogen bonding and π-π stacking between the aromatic groups of levofloxacin, both of which reduce the level of exposure of hydrophobic regions to the aqueous environment. Experimentally, this is seen in our NMR results, where levofloxacin shows concentration-dependent chemical shift changes and line broadening, indicative of dynamic self-association in solution (Figures 2 and 3). These spectral changes reflect the local clustering of the drug at the membrane interface or intercalation within the hydrophobic bilayer interior.

**Figure 6:**
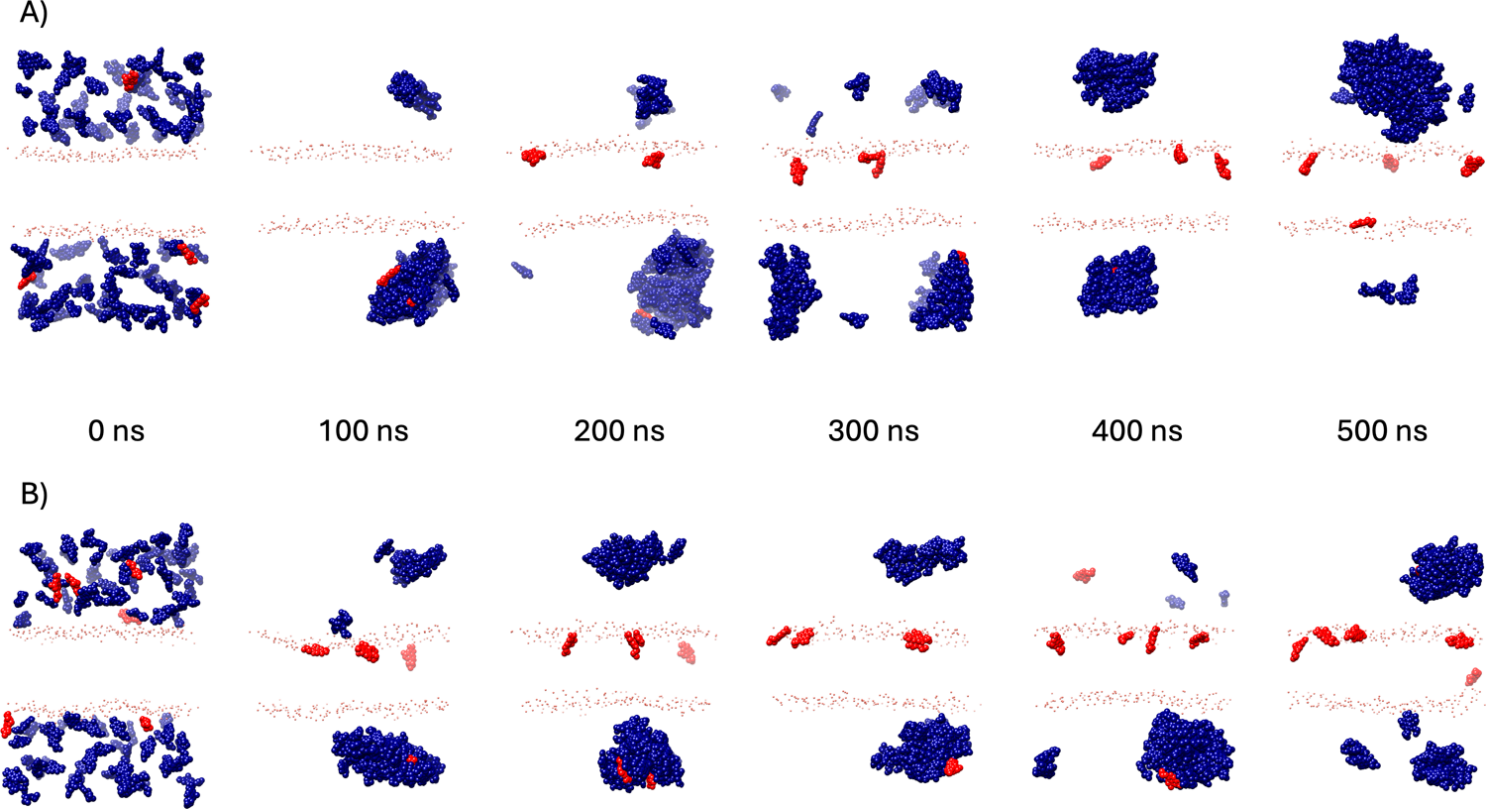
Atomistic MD simulations of levofloxacin reveal levofloxacin self-association in solution and partitioning into both zwitterionic membranes of (A) DOPC, and negative membranes of (B) DOPC/DOPG. Time frames are shown at 100 ns intervals from a 500 ns evolution trajectory of a 100 × 100 Å hydrated membrane patch of (A) DOPC or (B) DOPC/DOPG 3:1 with 100 levofloxacin molecules introduced from the solvent phase. Phosphorus atoms from the DOPC and DOPG headgroups are shown in orange. Levofloxacin molecules seen interacting with the membrane at any point in the trajectory are colored red, with the remaining shown in blue to highlight partitioning between membrane and solvent. In both trajectories (A) (DOPC) and (B) (DOPC/DOPG), levofloxacin molecules partition irreversibly into the lipid bilayer and do not rejoin the aqueous phase. Self-association of levofloxacin in solution occurs between 0 and 100 ns for both simulated membranes.

To understand the molecular details of levofloxacin interactions with negatively charged mixed lipid membranes, we carried out MD simulations of 100 levofloxacin molecules in the presence of a 100 × 100 Å membrane patch of 3:1 DOPC/DOPG. By the end of the 500 ns trajectory, five molecules have entered the membrane (Figure 6B). The 5% partitioning is below the 15% observation in our NMR study, which suggests that slow exchange and membrane insertion kinetics have not reached equilibrium during the 500 ns trajectory. Toward the end of the 500 ns trajectory, we observed an increase in local membrane curvature associated with levofloxacin insertion (Figure 6B, 500 ns frame), which could explain the small spectral contribution from a mobile lipid population in the wide-line ^31^P spectra of DOPC/DOPG/levofloxacin (Figure S1).

In both DOPC and DOPC/DOPG, the lipophilic to hydrophilic ratio was consistent with the known LogP of levofloxacin. As observed with the DOPC membrane, levofloxacin molecules that enter the membrane do not return to the aqueous phase. We did observe some levofloxacin molecules in exchange between droplets and the aqueous phase. This suggests that membrane integration is likely limited to monomeric levofloxacin, as self-associated multimers observed in the aqueous phase may be restricted from membrane insertion.

Similarities between levofloxacin interactions with zwitterionic and negative membranes are consistent with the lack of charge on the molecule. However, in acidic environments, protonation of the piperazine ring nitrogen in levofloxacin allows it to interact with negatively charged lipids.^48^ Despite this, the ability of the antibiotic to form hydrogen bonds is limited by its structure, with eight hydrogen bond acceptors and only one donor. This is likely to reduce the number of hydrogen bonds levofloxacin can form with nearby negatively charged lipids, which explains the similar interactions with zwitterionic and negatively charged membranes.

### DLS Reveals Liposome Integrity in the Presence of Levofloxacin

We used DLS to characterize the impact of levofloxacin interactions with membranes on membrane curvature and liposome morphology. The impact of levofloxacin concentration on DOPC and DOPC/DOPG liposome size was inferred from the mean diameter and polydispersity index (PDI) of liposomes (Table 1). The formation of larger liposomes broadly indicates a preference for more planar bilayers with a lower 2D curvature. We compare liposomes across a concentration range of levofloxacin between 0.5 and 40 mM DOPC, and between 5 and 40 mM for DOPC/DOPG, to assess the role of lipid composition and charge on membrane interactions with levofloxacin.

**Table 1:** Mean Diameter and Polydispersity of Unilamellar Vesicles with Various Concentrations of Levofloxacin Measured by Dynamic Light Scattering

	LFX concentration (mM)	Mean liposome diameter ± SD (nm)	Polydispersity index ± SD (PDI)
DOPC	0	404.3 ± 4.69	0.188 ± 0.025
	0.5	249.3 ± 2.19	0.187 ± 0.015
	1	248.1 ± 1.57	0.235 ± 0.004
	2	199.9 ± 1.91	0.178 ± 0.027
	5	216.0 ± 2.06	0.183 ± 0.014
	10	213.9 ± 2.09	0.166 ± 0.013
	20	227.2 ± 2.00	0.158 ± 0.015
	40	230.9 ± 1.64	0152 ± 0.014
DOPC/PG	0	243.1 ± 4.69	0.083 ± 0.003
	5	196.7 ± 0.77	0.165 ± 0.013
	10	197.6 ± 1.79	0.184 ± 0.015
	20	194.7 ± 1.39	0190 ± 0.012
	40	183.1 ± 4.06	0.197 ± 0.012

DOPC liposomes extruded through a 400 nm filter show close to true size (404.3 nm) in the absence of levofloxacin, while the mean liposome diameter ranged from 199 to 249 nm in the presence of levofloxacin. Liposomes consisting of DOPC showed a relatively homogeneous size distribution, while DOPC/DOPG liposomes were smaller and decreased in size from 196 nm at lower concentrations to 183 nm at the highest concentration of levofloxacin. The monodispersity in the size distribution across all concentrations remained good. Like DOPC, DOPC/DOPG liposomes in the absence of levofloxacin showed a larger homogeneous size distribution. However, at 243 nm, this indicates tighter lipid packing as opposed to DOPC alone. These results suggest that the presence of levofloxacin alters the structural properties of both membrane systems regardless of charge, possibly integrating into the membrane, altering lipid packing, and changing curvature that leads to smaller vesicles. Despite differences in final size, all liposome populations were monodisperse, suggesting that levofloxacin does not destabilize vesicles or cause aggregation or fragmentation.

These findings align with the chemical shift changes observed in NMR experiments, which demonstrated interactions between specific atoms of levofloxacin and membranes, dependent on lipid composition. The gradual reduction in size for DOPC/DOPG liposomes, coupled with their low polydispersity index, suggests that levofloxacin interacts more strongly and uniformly with bilayers containing the negatively charged DOPG. This supports the NMR data showing that polar regions of levofloxacin are more readily attracted to negatively charged bilayers and is consistent with the slightly larger membrane population of levofloxacin in DOPC/DOPG bilayers, seen in the MD simulations.

## Conclusion

The NMR observations are consistent with MD simulations, which show that levofloxacin inserts into the DOPC/DOPG membrane. In our simulations, 3/100 levofloxacin molecules insert into the bilayer within 100 ns, compared to 0/100 molecules entering the DOPC membrane (Figure 6). This suggests that the negatively charged DOPG initially favors levofloxacin insertion, particularly for the cationic piperazine ring. However, by 500 ns, 4/100 levofloxacin molecules enter the zwitterionic membrane, similar to 5/100 in the negatively charged bilayer. Simulations show that levofloxacin orients such that polar regions (hydroxyl, amine) are directed toward the lipid headgroups, while the hydrophobic fluoroquinolone core inserts into the acyl chain region.

Taken together, these data indicate that levofloxacin interacts more strongly and inserts more deeply into negatively charged membranes. The distinct, directional chemical shift changes across different nuclei provide insight into specific molecular interactions. Regions such as the piperazine ring and fluorine atom serve as sensitive reporters of membrane insertion, whereas aromatic protons H1 and H2 remain largely unaffected. Solution NMR thus offers high-resolution insight into membrane partitioning and self-association, with ssNMR and MD simulations confirming differences in the membrane binding and insertion depth.

The combined application of NMR characterization and MD simulations provides a comprehensive view of levofloxacin self-association and levofloxacin-membrane interactions. It reveals that levofloxacin undergoes concentration-dependent self-association in solution but reorients and partitions into lipid bilayers in a lipid-dependent manner. The ability to distinguish and characterize these discrete molecular states, free in solution, self-associated, or membrane-bound, offers mechanistic insight into levofloxacin’s physicochemical properties, membrane binding, and mode of action. These findings explain the diverse values of logP reported for levofloxacin and advance our understanding of how levofloxacin behaves in solution and in biologically relevant membrane environments to highlight the importance of drug-lipid interactions in modulating antibiotic activity. By resolving distinct molecular states and membrane insertion behaviors, this work provides a mechanistic basis for future efforts aimed at optimizing membrane permeability and improving the intracellular efficacy of fluoroquinolone-based therapeutics.

## Supplementary Material







## Notes

Molecular trajectory movies showing levofloxacin dynamics in the presence of DOPC and DOPC/DOPG membranes (PDF)
LFX100-DOPC (MP4)
LFX100-DOPCPG (MP4)

## Abbreviations

NMR: nuclear magnetic resonance
MD: molecular dynamics
DLS: dynamic light scattering
PDI: polydispersity index
DOPC: 1,2-Dioleoyl-*sn*-glycero-3-phosphatidyl choline
DOPG: 1,2-Dioleoyl-*sn*-glycero-3-phosphatidyl glycerol
MLVs: multilamellar vesicles
LUVs: large unilamellar vesicles
OmpC: outer membrane protein C
OmpF: outer membrane protein F
DMSO: dimethyl sulfoxide
ddH_2_O: double-distilled H_2_O
CS_iso_: isotropic chemical shift
CSA: chemical shift anisotropy
CP MAS: cross-polarization magic angle sample spinning
