# Supplementary material for: Molecular Interactions of Fluoroquinolone Antibiotics with Lipid Membranes
Source: Langmuir. 2025 Dec 2;42(6):4386–96. doi: 10.1021/acs.langmuir.5c04836 (PMC12921846; doi:10.1021/acs.langmuir.5c04836)
Supplement: Supplementary file 1 [file la5c04836_si_001.pdf]

Supporting Information

Molecular interactions of fluoroquinolone antibiotics with lipid membranes

By:

Dominic Ralph[1], Alice Goode[1], Vivien Yeh[1], Jessica Blair[2], Paul Williams[1] & Boyan B. Bonev[1]*

From the:

[1] School of Life Sciences, University of Nottingham, QMC, Nottingham NG7 2UH, UK

[2] Department of Microbes, Infection and Microbiomes, Institute of Microbiology and Infection, University of Birmingham, Institute of Microbiology and Infection, Birmingham, B15 2TT, UK

* Corresponding author

Table of Contents:

Supplemental figure S1. Phosphorous-31 wideline ssNMR spectra of DOPC and DOPC/DOPG (3:1) membranes without or with 40 mM levofloxacin recorded at 20°C.

Supplemental table S1. Phosphorous-31 MAS ssNMR longitudinal relaxation times T_1 from DOPC and DOPC/DOPG (3:1) membranes without or with 40 mM levofloxacin at 5 kHz MAS and 20°C.

Supplemental figure S2. Direct excitation natural abundance ^{13}C MAS ssNMR spectra of DOPC and DOPC/DOPG (3:1) membranes with 40 mM levofloxacin recorded at 5 kHz and 5°C.

Supplemental table S2: Resonance assignment from the ^{13}C CP MAS spectra of Figure 4 in the main manuscript. Levofloxacin resonances were assigned from solution NMR.

Supplemental figure S3: Trajectory movies over 500 ns of 100 levofloxacin molecules in the presence of a membrane patch of zwitterionic DOPC and negative mixed lipid DOPC/DOPG 3:1.

Keywords: fluoroquinolone antibiotics, lipid membranes, drug-membrane interactions, NMR, MD simulations

*Correspondence address: boyan.bonev@nottingham.ac.uk

To understand impact of levofloxacin on membrane phase stability and dynamics, we have carried out wideline ³¹P NMR study of DOPC and DOPC/DOPG 3:1 liposomes without and with 40 mM levofloxacin.

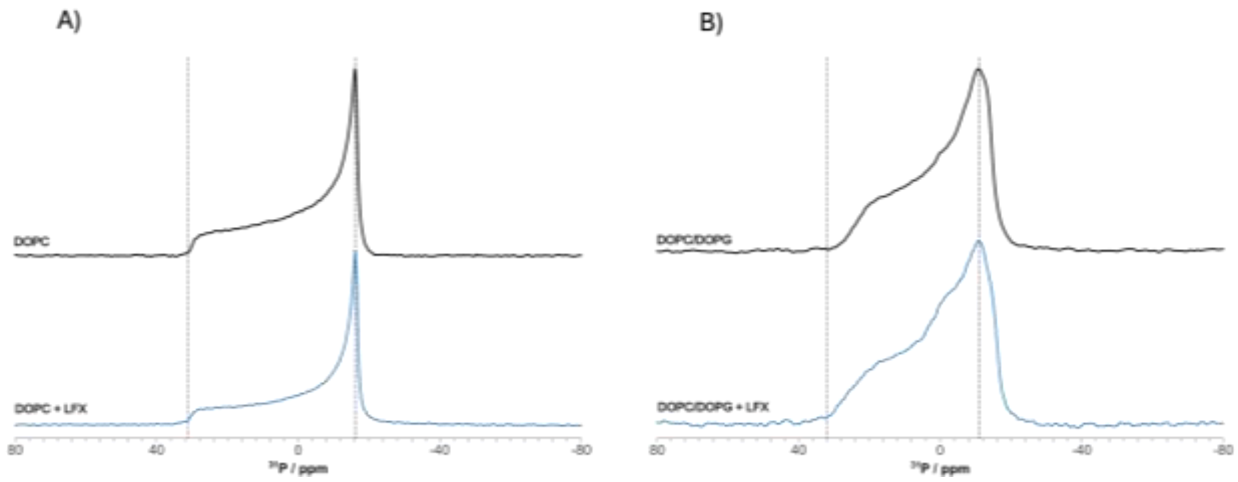

Supplemental figure S1. Phosphorous-31 wideline ssNMR spectra of (A) DOPC and (B) DOPC/DOPG (3:1) membranes without or with 40 mM levofloxacin recorded at 20°C. Dashed lines guide the eye to the 90 degree edges at -16 ppm and the 0 degree shoulders at 30 ppm of the Pake distribution. The DPOC/DOPG spectra show marked lateral oscillations, which round the characteristic features of the Pake patterns in B).

To understand the impact of levofloxacin membrane incorporation on fast lipid dynamics, we carried out longitudinal relaxation study by the inversion recovery method using ³¹P MAS NMR. The longitudinal relaxation times are summarised in Table ST1.

	T_1 (s) at 20°C	
	**DOPC**	**DOPG**
**DOPC**	0.78	-
**DOPC + LFX**	0.78	-
**DOPC/DOPG**	0.46	0.47
**DOPC/DOPG + LFX**	0.57	0.63

Supplemental Table S1: Phosphorous-31 MAS ssNMR longitudinal relaxation times T_1 from DOPC and DOPC/DOPG (3:1) membranes without or with 40 mM levofloxacin at 5 kHz MAS and 20°C.

In addition to the ^{13}C CP MAS NMR spectroscopy, reported in the main manuscript, we carried out direct excitation ^{13}C MAS NMR characterisation of the DOPC and DOPC/DOPG liposomes in the presence of levofloxacin. This approach reports ^{13}C levofloxacin resonances arising from carbons without directly attached protons, Figure S2. By contrast to CP MAS spectroscopy, direct excitation also reports ^{13}C NMR spectral intensity from levofloxacin that may have remained in the small amount of solution after collecting the lipid pellet by centrifugation.

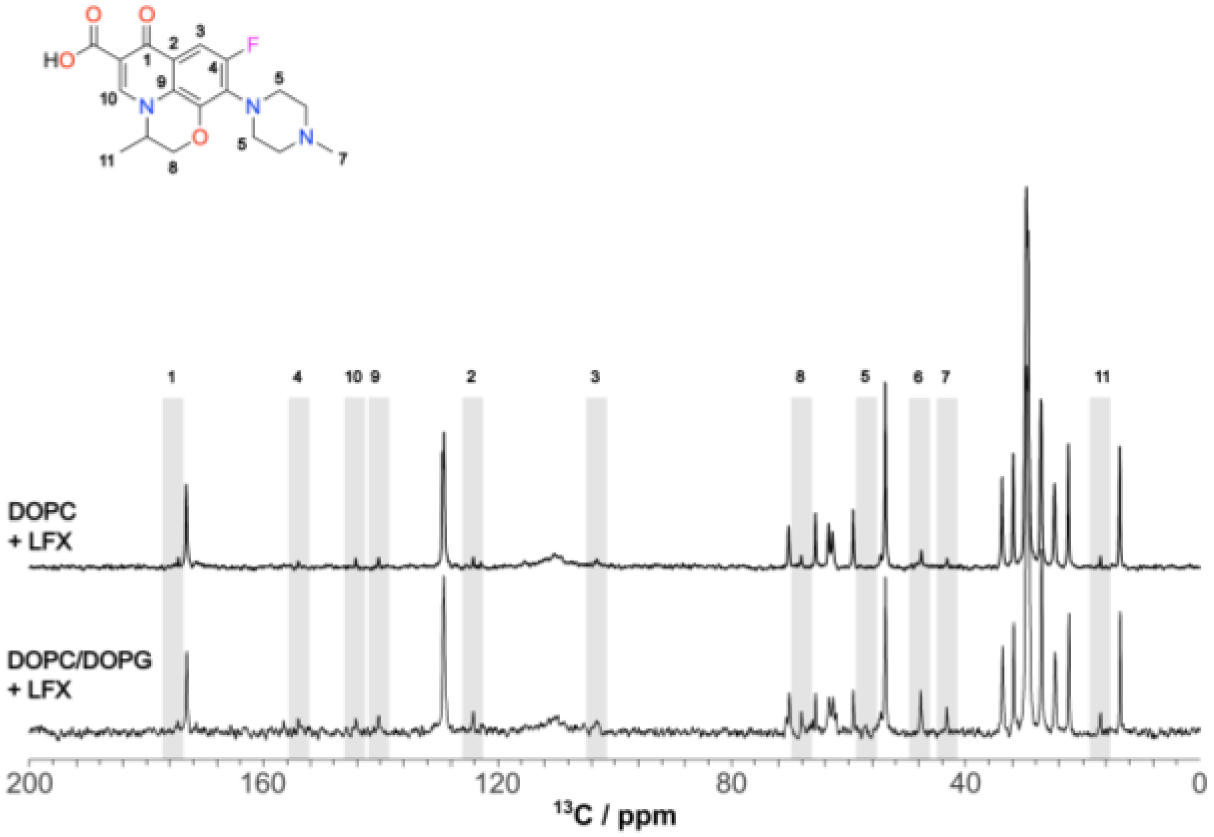

Supplemental figure S2. Direct excitation natural abundance ^{13}C MAS ssNMR spectra of (A) DOPC and (B) DOPC/DOPG (3:1) membranes with 40 mM levofloxacin recorded at 5 kHz and 5°C.

Assignment	PC/PG	PC/PG/LFX	PC	PC/LFX
CO	173.2	173.15	173.12	173.23
LFX1				144.2
C9	129.31	129.29	129.33	129.35
C10	129.1	129.13	129.08	129.12
LFX2		103.14		103.25
C2'	70.23	70.23	70.29	70.23
LFX3		68.14		68.12
Cb'	66.23	66.25		
Cb	65.66	65.72	65.69	65.7
C3'	63.4	63.43	63.36	63.42
C1'	62.71	62.7	62.74	62.76
Ca	59.23	59.24	59.17	59.22
LFX4				54.57
Cg	53.71	53.76	53.71	53.74
C2	33.7	33.71	33.71	33.73
C16	31.76	31.72	31.74	31.79
chain	29.48	29.29	29.5	29.52
C8-C11	27.06	27.03	27.05	27.08
C3'	24.7	24.71	24.71	24.74
C17	22.43	22.39	22.39	22.46
C18	13.65	13.62	13.59	13.68

Supplemental table S2: Resonance assignment from the 13C CP MAS spectra of Figure 4 in the main manuscript, following[1]. Levofloxacin resonances were assigned from solution NMR.

Supplemental MD trajectory movies

A B

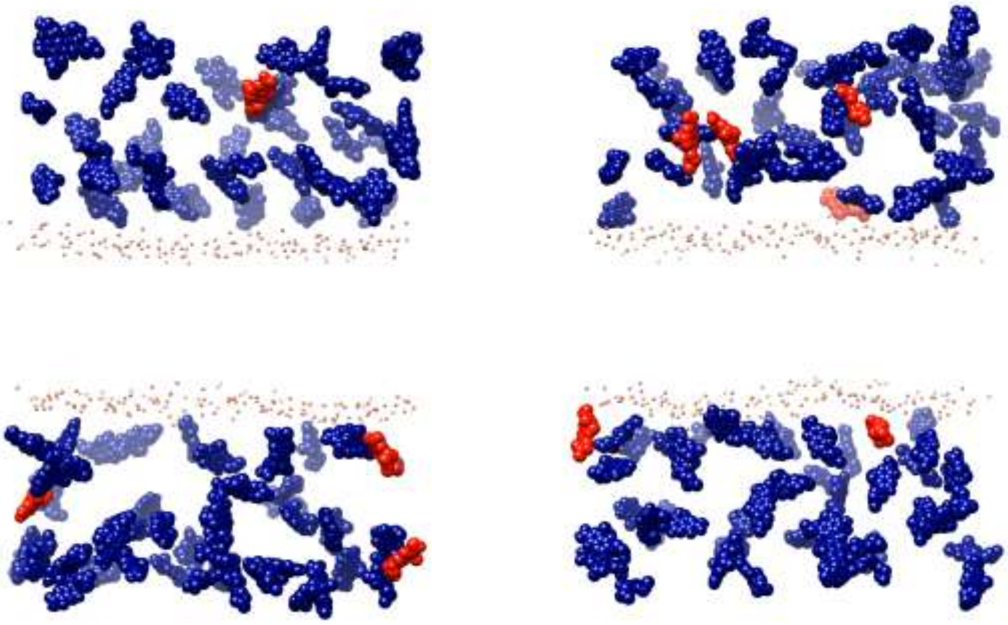

Supplemental figure S3: Trajectory movies over 500 ns of 100 levofloxacin molecules in the presence of a membrane patch of zwitterionic (A) DOPC and (B) negative mixed lipid DOPC/DOPG 3:1.

References

1. Zorin V, Ciesielski F, Griffin DC, Rittig M, Bonev BB. Heteronuclear chemical shift correlation and *J* -resolved MAS NMR spectroscopy of lipid membranes. Magnetic Resonance in Chemistry. 2010;48(12):925–34.
